# Of Good Mothers and Violent Fathers: Negotiating Child Protection Interventions in Abusive Relationships

**DOI:** 10.1177/10778012231158107

**Published:** 2023-02-22

**Authors:** Ella Kuskoff, Cameron Parsell, Stefanie Plage, Francisco Perales, Christine Ablaza

**Affiliations:** 1 School of Social Science, 1974 The University of Queensland, Brisbane, Queensland, Australia

**Keywords:** child protection, domestic violence, prevention, supportive housing for families

## Abstract

This article examines the efficacy of a supportive housing program aiming to provide mothers in violent relationships with the practical resources to minimize child protection intervention. Drawing on qualitative interviews with program mothers, child safety officers, and program practitioners, we explore the extent to which the program enabled mothers and children to live free from fathers’ violence and disengage from the child protection system. We find that, although valuable, the program did not fully mitigate the risks posed by violent fathers. We therefore argue that responsibility must be shifted onto violent fathers to change their behavior and build their parenting capacities.

## Introduction

Governments across the globe are increasingly recognizing and responding to the dangers of children being exposed to domestic violence (DV).^1^ DV—most often perpetrated by men against women—can have significant short- and long-term impacts on children and their development, even when the violence is not directed at them (Arai et al., 2021; Artz et al., 2014). Recognizing this risk, governments (including those in Australia, the United States, and the United Kingdom) are increasingly implementing strategies to protect children from the impacts of DV (see, e.g., Australian Government, 2021; Her Majesty’s Government, 2018; US Administration for Children and Families, 2019). In many cases, these strategies involve intervention by statutory child protection services (CPS) which, in serious cases, can result in children being removed from their parents and placed into the care of the state.

Although children have a right to live in a safe and violence-free environment (United Nations, 1989), feminist scholarship has long drawn our attention to the ways in which social structures and institutions can facilitate men's use of DV and further disempower women. A long-standing body of scholarship has examined how gendered power dynamics play out in legal systems (Bailey, 2010; Coker, 2001; De Simone & Heward-Belle, 2020; Douglas, 2018) and gendered policy responses (Abraham & Tastsoglou, 2016; Burnett et al., 2016; Hearn & McKie, 2010; Kuskoff & Parsell, 2020). Increasingly, research is also focusing on how gendered power dynamics manifest in CPS interventions (Azzopardi, 2021; De Simone & Heward-Belle, 2020; Douglas & Walsh, 2010; Maher et al., 2021). This literature highlights how CPS interventions are based on the assumption that mothers are to blame, attributing responsibility to mothers for the violence enacted by fathers, and placing undue burden on mothers to ensure children are not exposed to DV. Often, mothers are expected to end their relationship with fathers as a means of keeping their children safe (Meyer, 2011).

However, leaving a violent partner is a time of heightened risks for the safety of mothers and children (Douglas & Walsh, 2010). Expectations to leave have thus been heavily criticized by feminist scholars, both for overlooking these risks and for ignoring the many structural barriers that mothers face to safely and permanently ending relationships with their violent partners (Azzopardi, 2021; Kuskoff & Parsell, 2020; Maher et al., 2021; Meyer, 2011). These barriers include a lack of financial resources and access to affordable housing and have arguably been exacerbated by the COVID-19 pandemic (Pawson et al., 2021; United Nations, 2020). It is within the context of COVID-19 pandemic management that prominent multilateral organizations such as the United Nations have “urged all Governments to make the prevention and redress of violence against women a key part of their national response plans” (United Nations, 2020). This statement echoes the calls made by feminist scholars over recent years for increased investment in support to enable women to overcome the structural barriers to leaving violent relationships. Such supports are particularly important for mothers engaged with CPS, who face additional barriers to caring for and protecting their children in ways that satisfy the criteria codified in state child protection policies (Hughes et al., 2011; Lapierre, 2010; Meyer, 2011; Peled & Gil, 2011).

This article examines the efficacy of an intervention aiming to support mothers to minimize CPS involvement. The supportive housing for families (SHF) program pilot we examine was established in 2020 in the Australian state of Queensland, and explicitly recognizes and responds to the challenges faced by mothers with a history of experiencing DV and engagement with CPS. The pilot program aimed to provide mothers with the practical resources necessary to independently access secure and affordable housing and to care for and protect their children from violent fathers. For the purpose of our study, the term “father” is used to refer to men who play a fathering role to the children in the program, whether they be biological fathers, step-fathers, or a mother's romantic partner. Drawing on interviews with the mothers participating in the SHF pilot, statutory child safety officers (CSOs), and program support workers, this article aims to answer the following questions: (1) What were mothers' experiences of DV and CPS intervention prior to participating in the program? (2) How did mothers experience and leverage the support provided through the program? (3) How did mothers' experiences in the program influence their interactions with CPS? We discuss the implications of our findings for the delivery of support services moving forward.

## CPS Intervention in Families With Violent Fathers

### Child Protection Policy: Gendering the Blame

An enduring body of literature foregrounds the pervasiveness of gender-biased policies in statutory child protection systems (Azzopardi, 2021; Douglas & Walsh, 2010; Kitchen, 2014; Kopels & Sheridan, 2002). (Re)producing traditional gender ideologies surrounding caregiving roles and expectations, such policies are notorious for their over-emphasis on the responsibilities of mothers to care for their children, and their relatively limited engagement with the responsibilities of fathers (Azzopardi, 2021; De Simone & Heward-Belle, 2020). One notable and highly controversial form of policy in this regard is “Failure to Protect” policy (see, e.g., Azzopardi, 2021; Douglas & Walsh, 2010; Goodmark, 2010; Maher et al., 2021; Meyer, 2011). This form of policy positions parents as having an obligation to protect children from harm. Parents—most often mothers—who are unable or unwilling to provide such protection are seen as being responsible for the harm that they fail to prevent (Azzopardi, 2021). As Azzopardi (2021, p. 2) explains, “Women, mostly biological mothers, comprise 90% of those identified as primary caregivers in child maltreatment … With little purposeful engagement with fathers, caregiver capacity to protect tends to be synonymous with maternal capacity to protect” (Azzopardi, 2021, p. 2).

This unequal attribution of responsibility is highly problematic from a gender equity standpoint and has been linked to cultural ideals around the “good mother.” These ideals position women as individually responsible for their children's care and well-being. Moreover, the ideals necessitate that a mother wholeheartedly adopts the role of primary caregiver and nurturer of children, and foregoes her own needs to ensure the children's needs are met (Dunkerley, 2017; Maher et al., 2021; Peled & Gil, 2011). These expectations form an unrealistic baseline against which mothers engaged in child protection systems are assessed. Fathers, on the other hand, tend not to be held to the same high parenting standards and are often dismissed as playing an “optional” or secondary role in childrearing (Azzopardi, 2021; Cano et al., 2019; Peled & Gil, 2011).

Critically, “Failure to Protect” legislation becomes even more problematic when the threat mothers are expected to protect their children from is the children's father. Feminist scholarship is increasingly drawing our attention to the tendency of CPS policies to blame mothers for failing to protect their children from fathers' violent behavior and to attribute responsibility to mothers for preventing children's exposure to future violence (Azzopardi, 2021; De Simone & Heward-Belle, 2020; Douglas & Walsh, 2010; Goodmark, 2010). Indeed, when CPS becomes aware that children are at risk of being exposed to their father's DV, it is not uncommon for mothers to face the prospect of having their children removed from their care unless they end their relationship with the father (Douglas & Walsh, 2010; Maher et al., 2021; Meyer, 2011). Not only can ending a violent relationship put mothers and children at greater risk, but it also separates fathers from their parenting responsibilities and puts mothers in a difficult position should fathers attempt to maintain contact with their children. Fathers, on the other hand, are not often held accountable for their use of violence and the risks it poses to their children (Azzopardi, 2021), be that through the criminal justice system or through engagement in positive parenting education.

CPS policy that makes mothers responsible for protecting children from fathers' violent behavior—predominantly by ending their relationship—is not just problematic from a gender equity perspective; it is based on the erroneous assumption that mothers have the agency, opportunity, and power to do so. This assumption overlooks the multiple structural barriers and constraints on women's agency that prevent them from leaving or otherwise protecting their children from the father's violence (Azzopardi, 2021; Maher et al., 2021; Meyer, 2011). Rather than supporting mothers to access the resources necessary to safely disengage from violent fathers if this is their wish, or holding fathers to higher parenting standards, CPS interventions tend to focus on addressing mothers' perceived parenting deficiencies (Douglas & Walsh, 2010). In the following section, we discuss existing literature regarding how such interventions play out in practice.

### Child Protection Practice: Mothers as the Problem and the Solution

A small but increasing body of literature explores mothers' experiences with CPS intervention due to DV (Azzopardi, 2021; Hughes et al., 2011; Johnson & Sullivan, 2008; Maher et al., 2021; Meyer, 2011; Nixon et al., 2013; Stewart, 2021). This literature identifies four key themes, all of which serve to position mothers as both the problem and the solution to DV-related concerns. The first theme identified in the literature is the tendency for CPS to hold unrealistic expectations of mothers. This can include expectations to immediately and permanently end their relationship with the violent father, or risk having their children removed from their care (Azzopardi, 2021). Mothers can also face expectations to manage fathers' behavior, including by relaying CPS requirements to fathers (e.g., the requirement for fathers to contact CPS to provide certain information) and ensuring they comply. Such expectations are grounded in “good mother” ideals that prioritize the safety of children, often to the detriment of mothers' own safety. Despite CPS involvement rarely being their own fault, mothers are expected to uphold mothering standards that fail to account for the complex power dynamics that characterize DV and undermine their own needs and safety (Azzopardi, 2021; Douglas & Walsh, 2010; Dunkerley, 2017; Stewart, 2021).

The second theme in the literature is the tendency for CPS to burden mothers with responsibility for protecting children from violent fathers, while failing to engage with the violent fathers themselves. The literature shows that CPS risk assessments and care plans generally focus on what mothers must do to protect the child from the risk, while overlooking the need for fathers to end the risk by changing their behavior (Azzopardi, 2021; De Simone & Heward-Belle, 2020). This results in mothers being blamed for fathers' violence and facing stringent requirements to change their own behavior and improve their parenting skills, while the same is not true for fathers (Featherstone & Peckover, 2007; Johnson & Sullivan, 2008). Indeed, Azzopardi (2021) finds that although fathers tended to be the primary source of risk to their children, there is often limited contact between them and child safety workers. As fathers tend to refuse to engage with CPS, mothers are treated as “gatekeepers” to the family and have substantially more information recorded about them and their parenting practices than fathers. This helps to perpetuate CPS's focus on mothers' perceived deficiencies and responsibility to change.

The third theme identified in the literature relates to CPS's focus on mothers' deficiencies, and their tendency to overlook the various strategies mothers employ to keep their children safe. For example, although CPS often blames the mother for failing to leave violent fathers, research shows that mothers' decisions to leave or seek help are well considered and well informed by their assessments of what is best for their children at that point in time (Cramp & Zufferey, 2021; Douglas & Walsh, 2010; Maher et al., 2021; Meyer, 2011). Even when mothers feel it is not safe to leave or seek help, the literature indicates that they constantly employ strategies to minimize harm to their children. Such strategies range from attempting to predict violence and making sure children are not in the vicinity when it occurs, to taking the blame for their children's actions as a means of ensuring the violence is directed at them and not their children (Lapierre, 2010; Peled & Gil, 2011). These efforts to protect children are often overlooked by CPS, or dismissed as inadequate (Lapierre, 2010).

Finally, the fourth theme identified in the literature is the lack of practical support offered by CPS to enable mothers to enact any expected changes. Many existing studies highlight the numerous barriers that mothers face to living up to CPS requirements, particularly when those requirements involve ending their relationship with the violent father. These barriers include increased personal safety risks post-separation, financial barriers to leaving, and difficulties involved in having children named in DV protection orders (De Simone & Heward-Belle, 2020; Meyer, 2011). Despite these significant barriers, little support is provided to mothers to help overcome them (Meyer, 2011). As Azzopardi (2021, p. 22) argues, child protection policies and practices are characterized by “an unremitting expectation of immediate maternal readiness, willingness, and ability to forgo their own needs and overcome their own crises to fulfill onerous protection duties with little tangible assistance or clinical guidance.” This literature forms the basis of increasing calls for systemic change to better support the strengths of abused mothers, and to reorient CPS approaches to ensure the long-term safety and well-being of mothers and children (Lapierre, 2009, 2010; Meyer, 2011; Peled & Gil, 2011). In this article, we examine the efficacy of one program that aims to achieve this by providing mothers with the resources and support required to live independently of violent fathers and address CPS concerns.

## Queensland's Supportive Housing for Families Program

In 2020–2021, 54% of all Queensland households with a child deemed by CPS to be “at an unacceptable risk of significant future harm” and without “a parent willing and able to protect them” had experienced at least two episodes of DV in the preceding 12 months (Department of Children, Youth Justice and Multicultural Affairs, 2021a). This number is indicative of the significant role DV plays in families who are engaged with CPS. Importantly, the *Child Safety Practice Manual—Domestic and Family Violence* (Department of Children, Youth Justice and Multicultural Affairs, 2021b) explicitly recognizes and attempts to respond to many of the issues raised in the literature. According to the manual (p. 25), the removal of a child from an abused mother should only occur when “every reasonable attempt has been made to partner with the mother; and, every reasonable effort (across agencies and court systems) has been made to intervene with the perpetrator; and, when the perpetrator continues to have access to the children and presents an imminent risk to their safety.”

In 2020, the Queensland Government funded the SHF pilot program to help prevent the removal of children from low-income mothers and enable these mothers to disengage from CPS. The program aimed to provide the resources necessary for mothers to overcome structural barriers to live independently and maximize their agency to better care for and protect their children. The SHF program involved the provision of permanent and subsidized housing head-leased through the private rental market, as well as access to a range of supports tailored to the needs of each family (including parenting support, DV-specific support, and support to engage children in early childhood education).

The pilot was initially funded to support 20 families for a period of 12 months, but it was later extended to a period of 4 years. To be eligible to participate in the program, families had to have at least one child aged up to 5 years; be on extremely low income; be accessing homelessness support services; be involved with CPS or at risk of involvement; and be approved for social housing in Queensland.

The program design was informed by research demonstrating the significance of DV in the lives of CPS-involved mothers and thus strongly focused on supporting the safety of mothers experiencing DV. Of the participating mothers, 79% were single and 82% were either involved with CPS at the time or had previously been involved with them. Given existing knowledge regarding the high rates of DV among CPS-involved families and the highly gendered nature of CPS intervention, the SHF program provides a valuable opportunity to examine the extent to which a program targeted at supporting mothers to overcome structural barriers, such as access to affordable and secure housing, can enable said mothers and their children to live free from fathers' violence.

## Methods

Our study aims to answer three overarching research questions: (1) What were mothers' experiences of DV and CPS intervention prior to participating in the program? (2) How did mothers experience and leverage the support provided through the program? (3) How did mothers' experiences in the program influence their interactions with CPS? To answer these questions, we adopted a qualitative research methodology involving semi-structured interviews with mothers participating in the program, program support workers, and CSOs with mothers in the program on their caseloads. We invited all 20 mothers and 10 support workers involved in the program to participate in the research, as well as seven CSOs. A total of 17 mothers, 10 support workers, and 7 CSOs agreed to participate in interviews for the research. Given that there were a limited number of people involved in the SHF program, we did not seek to achieve saturation. Rather, data collection ceased when all potential participants we were able to identify and contact had been invited to participate in an interview.

Our institution's Human Research Ethics Committee reviewed and approved the research. We provided information about the purpose and expected commitments to prospective participants before obtaining their consent in writing. We developed an interview guide for mothers to explore their experiences with DV prior to and during the SHF program, as well as their expectations post-exit. Given the sensitive nature of the topic, questions and prompts were kept broad to allow mothers to exercise control over how much they wanted to share. For the most part, experiences with CPS interventions and DV were brought up by mothers themselves, rather than solicited by interviewers. A separate interview guide for CSOs and practitioners focused on their experiences with SHF program implementation and providing support to mothers aimed at minimizing CPS involvement.

All interviews were audio recorded, transcribed verbatim with participants' consent, and thematically analyzed (Padgett, 2017) using NVivo12 software. For our analysis, we took memos and notes during data collection, then read and re-read the transcripts and developed an initial coding frame. We discussed descriptive and analytical codes as a team and applied those to the transcripts. From this process, we developed themes that checked against findings reported in previous literature. Following Padgett (2017), we drew on several strategies to enhance the rigor of the study. For example, drawing on interviews with multiple groups who have complementary perspectives enabled us to triangulate participants' experiences. In doing so, we are able to provide a more comprehensive overview of the intersections between DV and CPS intervention. We also engaged the concept of reflexivity to constantly reflect on our positionality as researchers conducting a study involving mothers, CSOs, and support workers, and how this would bear on the analytical process (Lietz et al., 2006). It is not our intention to evaluate the presence of risks for mothers and their children but to understand the complementary perspectives and experiences of diverse participants.

The research on which the present article is based was conducted as part of a commissioned study on the SHF pilot. Data collection and an independent report were funded in part by the not-for-profit organizations delivering SHF pilot as well as philanthropic donations. No additional form of support was provided from these organizations, and the lines of inquiry pursued in our analyses here were undertaken independently of the original study.

## Findings

Below, we present our findings on mothers' experiences of DV as a factor motivating child protection intervention; mothers' experiences of support provided through the SHF program; and the implications for mothers' interactions with child protection. The aim is to examine the extent to which SHF enabled mothers and children to live free from fathers' violence and, in turn, disengage from the child protection system. We use illustrative excerpts from interviews using pseudonyms to support our conclusions.

### Mothers’ Experiences of Domestic Violence as a Factor Motivating Child Protection Intervention

Mothers' experiences with DV and its role in motivating CPS intervention were dominant in mothers' narratives. Prior to entering the program, mothers had experienced significant violence from one or more (ex-)partners, causing many to fear for their own safety and that of their children. For some mothers, DV and its impact on their children was the catalyst for them to end their relationship with a violent father, or to seek help from community or statutory services. As mothers Zoe and Isla recount:

He threatened to smack her, he was in her face yelling at her … She doesn’t need that. (Zoe, Mother)

When my ex-husband had custody of my three-year-old I didn’t like how he was treating him and everything like that, and so I called Child Safety and got involved with them. (Isla, Mother)

DV was also identified by mothers, support workers, and CSOs alike as a core reason for child protection intervention. As the following interview excerpts indicate, child protection concerns often centered on the risk posed by the father's use of violence:

[CPS] come in straight away when she was born … I was like, “Hold on a minute. [The father’s] not even here. He wasn’t even at the birth.” It was like, “Okay. Well, this is why we’re here.” And it wasn’t even about me, which was really frustrating … They took her straight off [me]. (Zoe, Mother)

She had a lot of involvement with Child Safety in the past due to sort of her mental health and previous domestic violence relationships that she's been in … We weren’t necessarily really investigating Mum, because it was about concerns about Dad's household. (Ava, CSO)

This risk was perceived by CSOs to be heightened by mothers' lack of access to safe and secure housing. As Charlotte and Ruby explain:

Housing is such a big issue … families can stay in an abusive relationship because they’ve got nowhere to go and their last resort is the refuge and they don’t want to go down that road. So they stay. (Charlotte, CSO)

A lot of the challenges that you have when you’ve got mothers that have left violent situations … is they don’t necessarily have that long term, safe housing. (Ruby, CSO)

The experiences of mothers in our sample reflect those identified in existing literature, whereby mothers are forced to navigate CPS interventions not due to their own behavior, but rather due to the violence of fathers (Azzopardi, 2021; De Simone & Heward-Belle, 2020; Douglas & Walsh, 2010; Maher et al., 2021). Perversely, women who experience DV can be deemed to have failed to protect their children because of fathers' violent behaviors. Furthermore, the literature, as well as the quotes above, make it clear that there are numerous barriers that make leaving violence difficult. These barriers not only include escalating violence and threats when women do leave (Douglas & Walsh, 2010; Meyer, 2011) but also material barriers such as insufficient income to live independently or difficulties accessing housing (Azzopardi, 2021; De Simone & Heward-Belle, 2020).

### Mothers’ Experiences of Supportive Housing for Families

Collectively, our interviews with mothers, support workers, and CSOs indicated that SHF was successful at supporting mothers to overcome structural housing barriers. Participants identified the program support workers' understanding of DV and the risks DV poses as a core factor contributing to this success. This understanding enabled the support workers to flexibly employ diverse strategies to protect the rights and safety of mothers:

She was escaping a domestic violence situation, and so [SHF] helped her find good housing that was secure and in a gated community … the housing has been very important for us because it's so secure for her. (Evie, CSO)

[SHF] recognised DV is present or is potentially present. They put the mum or the female of the relationship down as the approved tenant, and then they’ll add their male partners as an approved occupant. So, it's easier if there is an incident and they want to separate, it's easier to have the male removed and give the rights to the female. (Lily, Support worker)

Mothers described the feelings of safety and security that came with having access to safe and secure housing. As mothers Hazel, and Zoe explain:

With the whole housing stuff, honestly, it's kept away from the DV … now I’m in a stable house. (Hazel, Mother)

It's amazing, the security. I love the intercom system, because her dad does know where I live now … No one can get through the doors. I always keep that door locked. (Zoe, Mother)

These findings highlight the difference SHF has made in mothers' lives in terms of providing the safety and security that they required to live independently of violent fathers.

Despite these positive experiences, however, participants also raised significant areas in which the SHF program was inadequate to address the ongoing concerns women felt for their safety, and the ongoing concerns of CPS. Many of these concerns are related to the unpredictable and high-risk nature of DV. For instance, participants spoke about the risks posed to mothers when violent fathers found out their address. Support worker Hannah, for example, spoke of the fear that one mother felt when she began living alone with her children:

She's struggled, to begin with, to be in her own house and she's like, “I don’t know how to stay here. I’m scared. I’m scared.” Because she's been home invaded by one of her perpetrators before and bashed severely. (Hannah, Support worker)

Mother Hazel spoke of similar fears. Hazel was compelled to vacate her SHF home for fear of her own and her child's safety when her child's father discovered where she lived. As Hazel said:

I’m not living at home at the moment … There's actually DV concerns, where my eldest's dad knows where I am … [SHF] said they don’t have any information on what the goes are with the leases or where they could put me and whatnot until the lease ends. (Hazel, Mother)

Given that the house had been head-leased for 6 months and the program did not currently have access to any vacant properties, it was unable to rehouse Hazel to keep her safe. Hazel thus faced the prospect of paying out the lease of the house while couch surfing with friends, or facing a risk to her and her child's safety by returning to live in the house.

Significantly, Hazel already had a Domestic Violence Order (DVO) in place, which prevented the father from contacting her. This DVO was obtained with the support of the SHF program:

With my eldest's dad, yes [there is a DVO] … They’ve actually funded a lady … she helps with the court. (Hazel, Mother)

Despite having a DVO in place, however, Hazel still held significant concerns for her and her child's safety. Zoe had a similar experience, whereby her child's father discovered where she lived and continued to make attempts to contact and intimidate her even though there was a DVO in place:

[The SHF program] actually gave me a lawyer to call and helped with the DVO … That was great because I was so nervous. I was so scared. (Zoe, Mother)

He spotted me in [the local] shopping centre and he shoved letters in my mailbox. (Zoe, Mother)

Even though Hazel and Zoe appreciated the program's support to have DVOs put in place to help protect them from fathers' violence, these DVOs were unable to mitigate the risk posed by violent fathers, even in conjunction with secure housing. This suggests that even SHF's ability to work in tandem with broader protective legislative frameworks was insufficient to enable mothers to feel safe from the ongoing threat of fathers' violence.

Importantly, these are not limitations of the program as such, as a program could not feasibly be expected to change the high-risk nature of DV. Rather, these issues are indicative of the limitations that stem from a broader systemic focus on supporting mothers to keep themselves and their children safe, rather than holding fathers accountable for their behavior. We return to this point in our discussion.

### Implications for Mothers’ Interactions With Child Protection Services

The support SHF provided enabled some of the mothers to disengage from CPS. As CSOs Ava and Evie explained:

The main reason for closing off [our intervention] … the original worries weren’t even about [mum] to begin with … and because of the [SHF] support that was already wrapped around her. (Ava, CSO)

So if the mum is doing really well, but is surrounded well by good supports, then we feel comfortable stepping out of [the family's life]. (Evie, CSO)

For some mothers, then, SHF was able to achieve its overarching aim of providing the resources and supports necessary to help keep them safe from violent fathers and address CPS concerns.

However, even with the support of SHF, some mothers continued to face CPS intervention. In these situations, CPS appeared to continue to view children as being at risk of exposure to DV, even when mothers had a demonstrated history of protecting their children from fathers' violence. For example:

Often Child Safety, historic concerns of the family might be DV, and although there's housing and support and parenting support in place, that still can continue on. (Lily, Support worker)

I got really shitty there at one point with Child Safety. I was like, “Youse have nothing on me and she doesn’t have any contact with him at all.” He hasn’t seen her since she was six months old, almost a year-and-a-half, and I just want it to end. (Zoe, Mother)

These excerpts suggest that mothers continue to bear blame and stigma for fathers' past use of violence, even when they have ended the relationship. These mothers are ascribed the enduring label of “DV victims,” and have their future choices judged through this lens. Rather than focusing on the ongoing risk posed by fathers' violent behaviors, CPS focuses on mothers' previous “irresponsible” decisions to become involved with violent men. The assumption here is not that the risk to children stems from fathers, but rather from mothers' irresponsible relationship choices. This assumption provides limited scope for these mothers to disengage from the child protection system.

## Discussion

This study examined the efficacy of a supportive housing program aiming to provide mothers in violent relationships with the practical resources to minimize child protection intervention. Our findings resonate with calls from multilateral organizations and feminist scholars for greater support to enable mothers to overcome structural barriers and feel empowered to live and parent free from fathers' violence. Although the findings are based on the Queensland context and are not generalizable given our non-probability sample, they are nested within and contribute to existing research both within and outside of Australia. In particular, they contribute to a broader consensus around the importance of resources to enable mothers experiencing DV to overcome structural barriers to caring for their children, as well as the pressing need to engage fathers more deeply and constructively in parenting and child protection efforts. We discuss the significance of our findings for each of these bodies of literature in turn.

Our findings demonstrate that SHF was a valuable resource for participating mothers, as it provided them with the resources required to assert agency over their lives and the ability to live independently of violent fathers. This support played a significant role in the closure of CPS cases (Kuskoff et al., 2022). In contrast to CPS's tendency to expect mothers to forego their own needs and prioritize the needs of their children (Azzopardi, 2021; Douglas & Walsh, 2010; Dunkerley, 2017; Stewart, 2021), SHF helped fulfill mothers' needs to feel safe from violent fathers, which in turn enabled them to better protect and care for their children in line with CPS standards. Thus, the resources and security provided through SHF contributed to the disengagement of CPS from many mothers' lives. The provision of stable housing with in-built security measures, as well as multidisciplinary wraparound support, was crucial in achieving this outcome.

Despite the significant positive impacts of the SHF program and its capacity to mitigate some of the risks posed by violent fathers, our findings suggest that the program was unable to completely end fathers' violence, nor free mothers from the burden of protecting children from the violence. Indeed, mothers continued to carry the double burden of having to keep themselves and their children safe from a violent father, while continuing to raise and provide for their children as a single mother, often with continued CPS intervention. Escaping immediate violence, even in the presence of affordable housing and integrated support services, is not synonymous with living free from the threat of violence. This point is significant, given that mothers still face the prospect of having their children removed if “the perpetrator continues to have access to the children and presents an imminent risk to their safety” (Department of Children, Youth Justice and Multicultural Affairs, 2021b, p. 25).

We thus argue that heavily resourced SHF programs, as well as other interventions exclusively aimed at supporting mothers and their children, cannot singlehandedly address the threat of family violence and the sustained risk of profoundly traumatic statutory child protection intervention. This is not a limitation of these programs in and of themselves, as programs targeted at supporting mothers could not feasibly be expected to address all of the high and complex risks associated with DV. Rather, we take this as an indication that more must be done at the broader institutional level to shift the burden away from mothers being responsible for managing fathers' violence, and toward fathers being responsible for changing their violent behavior and building their capacities as parents.

A small body of literature considers how such a shift may be implemented in practice. This literature highlights the importance of foregrounding men's roles and responsibilities as fathers in effort to encourage them to change their violent behavior. Meyer (2018) and Smith and Humphreys (2019), for example, reported that men's identity as fathers and love for their children are crucial motivating factors for engaging in behavior-change programs. Similarly, Stanley et al. (2012) found that maintaining or regaining access to children was a motivating factor for fathers to initially engage with behavior-change programs. As fathers engaged with programs over time, an awareness of the impact of their behaviors and their desires to be better fathers helped motivate them to remain in the program and change their violent behaviors.

Our research also adds to the literature on the ongoing impacts of DV on women and children's lives, even after violent relationships are ended. Indeed, the very idea of “ending” a violent relationship with a child's father is problematic, as this does not mean a father ceases to be the child's parent. Even after separation, fathers have a parenting responsibility to their children and may wish to retain contact with their children. Mothers who have ended their intimate partner relationships with fathers must thus often still engage with them on matters relating to their children, exposing them to ongoing risks. This demonstrates that ending a domestically violent relationship is not the same as solving the harms caused by the violence. More must be done to hold fathers accountable for their violence and support them to change.

## Conclusion

In this study, we found that even in the presence of highly beneficial and valued affordable housing and linked support, women continued to be burdened by the behavior of violent fathers. On the one hand, women were burdened with managing their own and their children's safety from their former partner, especially when the former partner was the children's father. On the other hand, women were also responsible for the burden of keeping their children out of the children protection system, when in fact the risks of statutory child safety intervention had their roots in the father's violent behavior.

Based on these findings, we argue that, in addition to implementing programs like SHF that provide mothers with the resources and supports necessary to live independently, there is substantial scope to change how CPS and support sector organizations engage with fathers who use DV. More must be done to engage with fathers, both to appropriately apportion responsibility and—where possible—stop violence at the source. Importantly, holding fathers to account must not happen at the expense of supporting women to overcome structural inequalities; both responses must be implemented in tandem to ensure all mothers and fathers have access to the supports they need to be caring and protective parents.

## Notes

1. There is ongoing debate surrounding the strengths and limitations of the term “domestic violence.” Please see Kuskoff and Parsell (2021) for an overview of this debate, as well as the justification underpinning our use of the term.
